# Supplementary figures and images for: New Insight into the Related Candidate Genes and Molecular Regulatory Mechanisms of Waterlogging Tolerance in Tree Peony Paeonia ostii
Source: Plants (Basel). 2024 Nov 27;13(23):3324. doi: 10.3390/plants13233324 (PMC11644042; doi:10.3390/plants13233324)

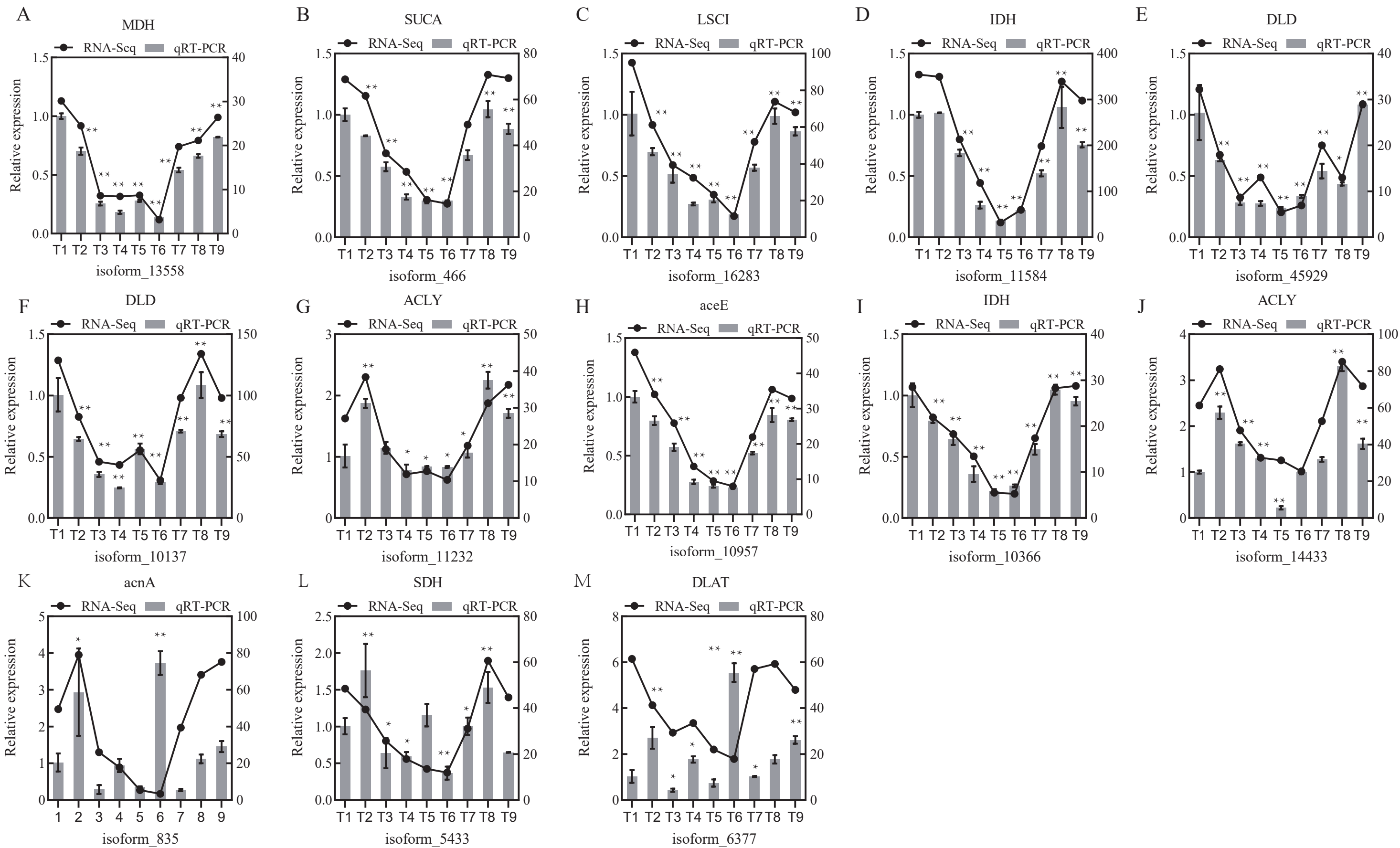

Supplement: Supplementary file 1 [file plants-13-03324-s001.zip › Figure S1-20241011.pdf]
